# The shadow challenges to improve the state essential newborn care practices in healthcare providers: evidence from a multicentre cross-sectional study in Ethiopia

**DOI:** 10.1186/s12887-021-02903-w

**Published:** 2021-10-07

**Authors:** Ermias Sisay Chanie, Amare Kassaw, Melkamu Senbeta, Fisha Alebel GebreEyesus, Aragaw Tesfaw, Abenezer Melkie, Tekalign Amera Birlie, Biruk Demissie, Demeke Mesfin Belay, Demewoze Kefale Mekone, Biniam Minuye Birhan, Wubet Alebachew Bayih

**Affiliations:** 1 Debre Tabor University, Debre Tabor, Ethiopia; 2 Assosa University, Assosa, Ethiopia; 3 Wolkitie University, Wolkitie, Ethiopia

**Keywords:** Essential newborn care practice, Ethiopia, Factors, Healthcare providers

## Abstract

**Background:**

Neonatal mortality can be reduced by providing essential newborn care. However, it is overlooked by most healthcare providers in Ethiopia. Hence, this study aims to examine immediate essential newborn care practices and associated factors among healthcare providers in Ethiopia.

**Methods:**

Institution-based cross-sectional study was conducted among 214 healthcare providers from November 11 to December 19, 2020, at a selected South Gondar health facility. Data were entered into Epi-data 4.2 and then exported to STATA14.0 for analysis. Both bivariable and multivariable logistic regression with a 95% confidence interval were computed. The variable that had a *p*-value less than 0.25 in bivariable logistic regression was entered into the multivariable logistic regression. In multivariable logistic regression, variables having a *p*-value < 0.05 were considered a statistically significant association with the poor practice of essential newborn care practice.

**Results:**

The overall essential newborn care practice among healthcare providers was found to be 74.8% (95% CI: 68.4, 80.2). Diploma educational status (AOR = 7.8, 95% CI:2.80–21.9), presence of workload (AOR = 9.7, 95% CI: 2.76–23.9), unavailability of drugs and vaccines (AOR = 9.8, 95% CI: 6.95–17.7), and having no training (AOR = 3.9, 95% CI: 1.73–8.92) were found to be predictors for poor essential newborn care practices.

**Conclusion:**

Essential newborn care practice among healthcare providers at South Gondar health institutions was found to be low. Being diploma educational status, presence of workload, unavailability of drugs and vaccines, and having no training were found to be independent predictors for poor practice of essential newborn care. Hence, periodic evaluation and strategies are needed for those predictor variables to address the gaps.

## Background

Essential newborn care is the care provided to the neonate after birth within the delivery room by skilled personnel, which includes drying and stimulating, assessing breathing, cord care, skin to skin contact, initiating exclusive breastfeeding, eye care, vitamin k administration, identification band, and weighing [1, 2].

Essential newborn care practices significantly reduce mortality and morbidity risk for the neonate, particularly for very small newborns [2–4]. Skilled care during labor can prevent about 50% of newborn mortality by reducing complications. Likewise, it can prevent 75% of newborn deaths in the postnatal period [5].

WHO planned to reduce neonatal deaths to below 12 per 1000 live births by 2030 [6], and approximately 70% of infant mortality occurs during the neonatal period [6]. Nevertheless, poor essential newborn care practice among healthcare providers, mainly in the resource-limited setting, is a great challenge to achieving the goal [5, 7, 8]. In addition, most healthcare providers give little attention, mainly in resource-limited settings [5, 9, 10].

Although the world health organization endorses improved ENC as a prioritized action around the time of birth to reduce neonatal mortality significantly [11], most healthcare providers do not practice appropriately [12]. Many neonatal mortalities can be reduced by providing essential newborn care. However, it is overlooked by most healthcare providers in Ethiopia [13].

Although different strategies have been implemented to enhance essential newborn care, only 16.4 and 13% of newborns obtained skilled health workers during delivery and the postnatal period respectively [14].

Neonatal mortality is unacceptably high at the time of birth in Ethiopia. However, the state of essential newborn care practice among healthcare providers is not well explored in Ethiopia in general and the study area in particular. Hence, this study aims to assess the immediate essential newborn care practices and associated factors among healthcare providers in South Gondar hospitals, Northwest Ethiopia, in 2021.

## Methods

### Study setting and period

The study was conducted at South Gondar hospitals from November 11 to December 19, 2020.

The South Gondar zone hospitals comprise one referral hospital and seven primary hospitals.

There are a total of 717 health care providers providing different services, such as inpatient and outpatient, and neonatal intensive care units. From the total of healthcare providers, around 397 healthcare providers were midwifery and nurses, according to the South Gondar administrator.

### Study design

A multicenter institution-based cross-sectional study was conducted.

### Study population

All healthcare providers who work in South Gondar hospitals.

### Inclusion criteria and exclusion criteria

All healthcare providers who work in selected South Gondar hospitals were eligible for the study. Whereas, healthcare providers who are seriously ill and on annual leave were excluded from the study.

### Sample size determination and sampling procedures

A single population proportion formula was used to estimate the sample size and the following.

assumptions were made p = proportion of essential newborn care practice of healthcare providers was 72.77% (*p* = 0.7277) from the previous study [10], level of significance 5% (α = 0.05), 95% confidence level (Z α/2 = 1.96) and absolute precision or margin of error 5% (d = 0.05). Where, *n* = sample size, Z = standard normal distribution curve value for the 95% confidence interval (1.96), d = the margin of error or accepted error, *n* = 305 health care providers. Adding a 10% allowance for a non-response rate, the total sample size was 336. But the total number of health professionals at the selected South Gondar health facilities was 214, therefore we included all of them.

There are a total of 8 hospitals in the South Gondar Zone. The hospitals were categorized into referral hospitals and district hospitals strata. Then, from the primary hospitals’ stratum, Addis zemen and Mekan Eyesus, Nefas mucha primary hospitals were selected randomly. From the referral hospital stratum, Debre Tabor referral hospital was selected since it is the only referral hospital in the South Gondar Zone. Then, all midwives and nurses in the selected health institution were included.

### Operation definition

There were nineteen procedures to assess the immediate newborn care practice through an observation checklist. Then the outcome variable was dichotomized into good practice or poor practice of immediate newborn care practice.

We used the term shadow or hidden observation, which means that the problems are not easy to notice or discover in the study area. This helps stakeholders to find a meaningful opportunity to improve essential newborn care practices, and take insight-driven action to create change in their setting.

Good practice: if the health care providers perform more than or equal to 70% of the practice procedures [10].

Poor practice: if the health care providers perform less than 70% of the practice procedures [10].

### Data collection procedures and quality control

The data were collected by four healthcare providers through a pre-tested observation checklist. The checklist comprised socio-demographic and clinical related characteristics. Moreover, data collectors were provided the purpose of the study to each study participant before the time of data collection. The validity of the checklist was ensured by developing different types of articles. The pretest was conducted in 10% of the calculated sample size in another health institution which was not included in the study. Two-day training and orientation were provided about the process of data collection for data collectors and supervisors. Moreover, the filled formats were checked for completeness by the supervisor, data cleaning, and double data were carried out to check for any inconsistencies, coding errors, missing values, and out of range daily.

### Data processing and analysis

Data were entered into Epi-data V. 4.2 and exported to STATAV.14.0 for analysis. Nineteen (19) standard checklists were prepared to assess the practice of essential newborn care. The descriptive data were explored through mean, standard deviation, and tables. Both bivariable and multivariable logistic regression with a 95% confidence interval were computed to identify the associated explanatory variable. The variable that had a *p*-value of less than 0.25 in the bivariable logistic regression was entered into the multivariable logistic regression. In multivariable logistic regression, variables having a *p*-value < 0.05 were considered a statistically significant association with poor practice of immediate essential newborn care practice. Multi-collinearity between the study variables was first diagnosed using the standard error and correlation matrix. Besides, Hosmer-Lemeshow statistics and Omnibus tests were performed, and Hosmer-Lemeshow’s test was found to be insignificant (*p*-value = 0.29). Additionally, Omnibus tests were significant (*p* ≤ 0.01) indicating the model was fitted.

## Results

### Socio-demographic characteristics of healthcare providers

Out of 214 healthcare providers, 119 (55.6%) were female. The majority of 166 (77.57%) healthcare providers were orthodox. Likewise, 179 (83.64%) of healthcare providers have a bachelor’s degree or above. Ninety-nine (46.26%) of healthcare providers were age between 25 and 29 years, and 121 (56.54%) were married.

From the total 214 healthcare providers, 136 (63.55%), 86 (40.19%), and 119 (55.61%) were nurses, monthly salary of between 5000 and 6500 Ethiopian birr, and 0–5 years of working experience respectively. One hundred eight (59.81%) of healthcare providers had a workload, whereas 131 (61.21%) of healthcare providers obtained training about immediate essential newborn care practice. Furthermore, 151 (70.56%) and 162 (75.70%) of the healthcare providers informed us that there was available equipment and drugs/vaccines in their working areas respectively (Table 1).

**Table 1:** Socio-demographic characteristics of healthcare providers in South Gondar hospitals, Northwest Ethiopia, 2021 (*n* = 214)

Variable	Frequency	Percent
Age		
1. 20–24	19	8.88
2. 25–29	99	46.26
3. 30–35	77	35.98
4. > 35	19	8.88
Sex		
1. Male	95	44.39
2. Female	119	55.61
Religion		
1. Orthodox	179	83.64
2. Muslim	26	12.15
3. Protestant	9	4.21
Educational status		
1. Degree	166	77.57
2. Diploma	48	22.43
Marital status		
1. Single	76	35.51
2. Married	121	56.54
3. Divorced	17	7.94
Field of study		
1. Nurse	136	63.55
2. Midwifery	78	36.45
Monthly salary		
1. 2000–3500	18	8.41
2. 3500–5000	33	15.42
3. 5000–6500	86	40.19
4. > 6500	77	35.98
Working experience (in the year)		
1. 0–5 years	119	55.61
2. 6–10 years	66	30.84
3. > 10 years	29	13.55
Workload		
1. Yes	128	59.81
2. No	86	40.19
Training on immediate newborn care		
1. Yes	131	61.21
2. No	83	38.79
Availability of equipment		
1. Yes	151	70.56
2. No	63	29.44
Availability of drugs and vaccines		
1. Yes	162	75.70
2. No	52	24.30

### The practice of essential newborn care among healthcare providers

From the total 214 healthcare providers, 165 (77.10%), 170 (79.44%), 174 (81.31%), and 121 (56.54%) were performed hand washing before the procedure, wearing a sterile glove, wearing an apron, and wearing a mask respectively.

The majority of 168 (78.50%),193 (90.19%), 175 (81.78%), and 181 (84.58%) of healthcare providers have wiped the eyes faces when the head is delivered, dry the baby immediately with a dry towel, Check & sucks the airway after delivery, and take APGAR score respectively. Besides, a large proportion of 171 (79.91%) healthcare providers performed umbilical cord care properly. A large proportion of 187 (87.38%), 137 (64.02%), and 195 (91.12%) of healthcare providers initiated breastfeeding within the first hour of delivery, counseled mothers about new bore danger before discharge, and weighed & recorded the baby’s weight respectively. Similarly, 173 (80.84%) of healthcare providers performed skin to skin contact. One hundred forty-three (66.82%) healthcare providers were administered Vitamin K, and 145 (67.76%) healthcare providers were given eye ointment for neonates (Table 2).

**Table 2:** Clinical related characteristics of healthcare providers in South Gondar hospitals, Northwest Ethiopia, 2021 (*n* = 214)

Variable	Frequency	Percent
Hand washing before the procedure		
1. Yes, performed	165	77.10
2. No, never	49	22.90
Put on a sterile glove		
1. Yes, performed	170	79.44
2. No, never	44	20.56
Wearing apron		
1. Yes, performed	174	81.31
2. No, never	40	18.69
Wearing mask		
1. Yes, performed	121	56.54
2. No, never	93	43.46
Wipe the eye &face when the head is delivered		
1. Yes, performed	168	78.50
2. No, never	46	21.50
Dry the baby immediately with a dry towel		
1. Yes, performed	193	90.19
2. No, never	21	9.81
Check & sucks the airway after delivery		
1. Yes, performed	175	81.78
2. No, never	39	18.22
Take APGAR score		
1. Yes, performed	181	84.58
2. No, never	33	15.42
Umbilical cord care		
1. Yes, performed	171	79.91
2. No, never	43	20.09
Skin to skin contact		
1. Yes, performed	173	80.84
2. No, never	41	19.16
Initiate breastfeeding within the first hour of delivery		
1. Yes, performed	187	87.38
2. No, never	27	12.62
Administer Vit K		
1. Yes, performed	143	66.82
2. No, never	71	33.18
Give eye ointment		
1. Yes, performed	145	67.76
2. No, never	69	32.24
Counsel mother about new bore danger before discharge		
1. Yes, performed	137	64.02
2. No, never	77	35.98
Weigh & record the baby’s weight		
1. Yes, performed	195	91.12
2. No, never	19	8.88

### Factors that affect the practice of immediate newborn care among healthcare providers

In bivariate logistic regression analysis, sex, educational status, the field of study, working experience, monthly salary, workload, and availability of drugs and vaccines variables were having *P*-value < 0.25 and entered into multivariable logistic regression.

In multivariable logistic regression, male, diploma educational status, presence of workload, and unavailability of drugs and vaccines were found to be predictors of the poor practice of essential newborn care.

The odds of poor practice essential newborn care among diploma healthcare providers were 3.0 times higher as compared to degree or above healthcare providers (AOR = 3.0, 95% CI: 12.8–71.8). Moreover, the odds of poor practice of essential newborn care among healthcare providers who had a workload were 2.9 times higher for the poor practice of essential newborn care than among healthcare providers who hadn’t a workload (AOR = 2.9, 95% CI: 1.18–7.27). The healthcare providers who were working on unavailability drugs and vaccines were 5.5 times higher for the poor practice of essential newborn care than the healthcare providers who were working on availability drugs and vaccines (AOR = 5.5, 95% CI: 2.20–13.8). Likewise, the healthcare providers who hadn’t taken essential newborn care training were 3.9 times higher for the poor practice of essential newborn care than the healthcare providers who had taken the training (AOR = 3.9, 95% CI: 1.73–8.92) (Table 3).

**Table 3:** Bivariable and multivariable logistic regression of healthcare providers in South Gondar hospitals, Northwest Ethiopia, 2021 (*n* = 214)

ENBC practice			OR (95% CI)		
	Good (160)	Poor (54)	COR	AOR	*P*-value
Age					
20–24	14	5	0.77 (0.19–3.16)		
35–29	78	21	0.58 (0.19–1.72)		
30–35	55	22	0.87 (0.29–2.57)		
> 35	13	6	1		
Sex					
Male	64	31	0.5 (0.25–0.93)	0.5 (0.22–1.13)	0.096
Female	96	23	1	1	
Religion					
Orthodox	133	46	1.2 (0.24–6.04)	–	
Muslim	20	6	1.1 (0.17–6.46)	–	
Protestant	7	2	1		
Educational status					
Diploma	22	26	**3.5 (1.78–7.04)**	**3.0 (1.28–7.18)**	0.012*
Degree and Above	138	28	1	1	
Marital status					
Single	57	19	1.6 (0.40–6.00)	–	
Married	89	32	1.7 (0.45–6.22)	–	
Divorced/Widowed	14	3	1		
Field of study					
Nurse	108	28	1	1	
Midwifery	52	26	1.9 (1.02–3.61)	1.2 (0.56–2.73)	0.600
Monthly salary					
2000–3500	11	7	2.1 (0.71–6.17)	1.2 (0.30–4.78)	0.803
3500–500	27	6	0.7 (0.26–2.04)	0.7 (0.19–2.30)	0.509
5000–6500	63	23	1.2 (0.59–2.44)	0.8 (0.34–2.08)	0.708
> 6500	59	18	1	1	
Working experience (in the year)					
0–5 years	73	46	3.9 (1.29–12.0)	3.6 (0.96–13.1)	0.051
6–10 years	62	4	0.4 (0.09–1.74)	0.4 (0.07–1.75)	0.203
> 10 years	25	4	1	1	
Workload					
Yes	82	46	**4.6 (2.13–10.1)**	**2.9 (1.18–7.27)**	0.021*
No	78	8	1	1	
Training					
Yes	100	31	1	–	
No	60	23	**3.1 (1.64–5.88)**	**3.9 (1.73–8.92)**	0.001**
Availability of equipment					
Yes	116	35	1	–	
No	44	19	1.4 (0.74–2.76)		
Availability of drugs and vaccines					
Yes	131	31	1	1	
No	29	23	**2.5 (1.30–4.98**	**5.5 (2.20–13.8)**	0.000**

* Significant at < 0.05; ** Significant at < 0.01; COR Crude odds ratio, *AOR* Adjusted odds ratio, *CI* Confidence interval, *ENBC* Essential new born care practices

## Discussion

The overall essential newborn care practice among healthcare providers at South Gondar health facility was found to be 74.8% (95% CI: 68.4, 80.2). This finding is consistent with another study conducted in Tigray Ethiopia, 72.77% [10] and Addis Ababa Ethiopia, 80.7% [15]. However, the finding is higher than the study conducted in Uganda 46.5% [16], Sudan41.1% [17], Vietnam 64% [18], Egypt 69.2% [19], Tigray Ethiopia 59.8% [6], and Afar Ethiopia (62.7%) [14].

The difference might be due to variation in accessibility of materials and study participants. Since most of the above studies included all healthcare providers, whereas only nurses and midwives were included in our study. Moreover, the study period can also contribute to the difference because the quality of essential newborn care practice has increased over time.

The odds of poor practice essential newborn care among diploma healthcare providers were 3.0 times higher as compared to degree or above healthcare providers. This finding is consistent with another setting in Ethiopia [5, 9]. This can be explained by the healthcare provider having a higher level of education status can be recognized and manage more common health problems in newborns.

The possible explanation might be that the high educational level of healthcare providers might generally have greater decision-making power and skill regarding the implementation of essential newborn care. Besides, healthcare providers who have a higher level of education might have a chance to obtain different kinds of training and skills that bring good practice in essential newborn care.

The healthcare providers who had a workload were 2.9 times higher for the poor practice of essential newborn care than healthcare providers who hadn’t workload. This is a finding supported by another setting in Ethiopia [6, 14]. In fact, healthcare providers with a workload, their performance are diminished because they may not have sufficient time to perform tasks that can have a direct effect on the quality of care. Besides, heavy healthcare provider workload can influence the care provider’s decision to perform various procedures and, it adversely affects patient safety [20, 21].

The healthcare providers who were working on unavailability of drugs and vaccines were 5.5 times higher for the poor practice of essential newborn care than the healthcare providers who were working on availability drugs and vaccines. This finding is supported in another setting [6, 9, 10]. Even if the healthcare providers have adequate knowledge and skills regarding essential newborn care practice, they might be unable to provide the service due to lack of materials. Hence, ensuring the essential drugs and vaccines is crucial to improving neonatal health and ultimately decreasing neonatal mortality.

Likewise, healthcare providers who hadn’t taken essential newborn care training were 3.9 times higher for the poor practice of essential newborn care than the healthcare providers who took the training. This finding is congruent with the previous studies [5, 17]. This can be explained by healthcare providers who took essential newborn care training or courses that can ensure the skills and knowledge to provide up-to-date evidence-based information and management for a range of needs in the initial newborn period [22–24].

This study has some limitations. First, this study does have inherent limitations due to the cross-sectional nature of the study, which used a snapshot of assessing essential newborn care practice at one point in time. Secondly, data were collected from midwives and nurse health care providers only that might be over or under the level of essential newborn practice.

## Conclusion

Essential newborn care practice among healthcare providers at South Gondar health institutions was found to be low. Being diploma educational status, presence of workload, unavailability of drugs and vaccines, and having no training were found to be independent predictors for poor practice of essential newborn care. Hence, periodic evaluation and strategies are needed for those predictor variables to address the gaps.

## Data Availability

The data will be available upon request from the corresponding author.
